# Advanced Materials for Solar Energy Utilization

**DOI:** 10.3390/ma18153511

**Published:** 2025-07-26

**Authors:** Xingwang Zhu, Tongming Su

**Affiliations:** 1 School of Environmental Science and Engineering, Institute of Technology for Carbon Neutralization, Yangzhou University, Yangzhou 225009, China; 2 School of Chemistry and Chemical Engineering, Guangxi Colleges and Universities Key Laboratory of New Technology and Application in Resource Chemical Engineering, Guangxi University, Nanning 530004, China

In the context of sustainable societal development, exploring novel, environmentally sustainable energy sources has emerged as a pivotal global concern [1,2,3]. As an inexhaustible, environmentally friendly, renewable and clean energy source, solar energy has been studied by a wide range of researchers [4,5,6,7,8]. Photocatalytic technology is predicated on advanced light-conversion materials, which efficiently drive the conversion of solar into chemical energy. They generate active species under light to degrade pollutants [9,10,11,12], convert energy [13,14,15,16,17], pursue environmental remediation [18,19,20,21], etc., so as to promote green industry chain development.

This Special Issue comprises a total of eleven original research articles authored by scientists from a variety of international backgrounds. The research presented here focuses on photocatalytic carbon dioxide (CO_2_) reduction, photocatalytic hydrogen production from water decomposition, the development of new and advanced photocatalytic materials, photocatalytic pollutant degradation, the preparation of photovoltaic materials, and photoelectron catalytic oxidation. This Special Issue provides a platform for scientists to present their original research on “Advanced Materials for Solar Energy Utilization”. The following brief synopses outline the papers that we have been honored to include, with the aim of highlighting advanced materials that have recently enabled solar energy conversion for use.

The substantial discharge of industrial CO_2_ has been identified as pivotal in the genesis of numerous ecological issues, with the mitigation of CO_2_ through photocatalysis emerging as a compelling technological solution in addressing the prevailing challenge of carbon emissions. Xu et al. [22] synthesized oxygen vacancy-modified MIL-125(Ti) with a high specific surface area via calcination in a hydrogen–argon mixture and applied it in photocatalytic CO_2_ conversion. The introduction of oxygen vacancies (OVs) resulted in an increase in the surface area and internal pores of the material, as well as in the Ti^3+^/Ti^4+^ ratio. The presence of OVs facilitated electron transfer from the Ti sites on MIL-125(Ti) to CO_2_, while lowering its activation energy barrier. The results demonstrate that the OV-modified MIL-125(Ti) performs exceptionally, at almost 100% selectivity in photocatalytic CO_2_ reduction.

The efficiency of visible light-driven photocatalytic CO_2_ reduction is relatively low when photogenerated carriers in photocatalysts are recombined. Li et al. [23] prepared WO_3_/BiOBr composite catalysts with fast charge separation performance to increase the efficiency of photogenerated carrier separation. This was attributed to the formation of a compact S-scheme heterojunction between WO_3_ and BiOBr, which has been demonstrated to accelerate photogenerated charge separation. The yield of CO_2_ photoreduced to CO by WO_3_/BiOBr was found to be 1.56 times higher than that of BiOBr in the visible light range and without the assistance of a sacrificial agent.

Crystalline phase engineering represents a highly efficacious method for modifying electron transfer pathways and regulating the internal electronic structure and separation efficiency of electron photocatalysts. Sun et al. [24] prepared Ru cocatalysts with face-centered cubic (fcc) and hexagonal close-packed (hcp) structures, modifying C_3_N_4_ by precisely adjusting the ratio of the Ru precursor and reducing solvent. Compared with fcc-Ru, hcp-Ru/C_3_N_4_ exhibited superior charge separation and transfer efficiency. Moreover, Gibbs free energy calculations demonstrated that the hydrogen adsorption energy (ΔG_H*_ = −0.14 eV) of hcp-Ru was more closely aligned with the optimal value than that of fcc-Ru (−0.32 eV). The process of hydrogen production by hydrolysis achieved through the utilization of hcp-Ru has been demonstrated to be both efficient and highly stable.

Chalcogenide quantum dots have been shown to exhibit fluorescence properties due to their ultrasmall size, with surface and quantum confinement effects being the main factors in this regard. Li et al. [25] successfully prepared uniformly dispersed and size-tunable CsPbBr_3_ QDs by changing the halogens in metal halide chalcogenides on the basis of the theory of LaMer nucleation and growth, and creating a Br^−^-rich reaction environment using the thermal injection method. This study demonstrated that CsPbBr_3_ QDs exhibit specific optical properties when their size reaches the critical Bohr exciton radius value. A further decrease in size results in a photoluminescence wavelength shift to the deep blue band, as well as the manifestation of distinct fluorescence properties. This creates potential for the construction of novel composite catalysts for applications in photocatalysis.

The extensive utilization of antibiotics has the potential to compromise ecological balance and human health. Consequently, researchers have explored the photocatalytic degradation of antibiotics, a process that offers numerous environmental advantages, among others. Su et al. [26] synthesized a Cd_x_Mn_1−x_S solid solution with precise control over the ratio of Mn^2+^ and Cd^2+^ ions and prepared C@Cd_x_Mn_1−x_S composite catalysts by compositing Cd_x_Mn_1−x_S with biomass-gasified carbon slag using a hydrothermal method. The active species of •O_2_^−^ and H^+^ produced in C@Cd_x_Mn_1−x_S were directly involved in the photocatalytic degradation reactions of tetracycline (TC), with the degradation efficiency reaching 90.35% within 60 min.

Wang et al. [27] prepared an environmentally friendly polyacrylonitrile (PAN)-based Janus-structured composite membrane by adjusting the asymmetric wettability through electrostatic spinning; they improved the thickness of both by employing TiO_2_-modified PAN as a hydrophilic base layer and PCL as a hydrophobic layer. The prepared PAN/TiO_2_-PCL20 composite membranes demonstrated optimal oil–water separation performance for oil emulsions stabilized with various surfactants. In addition, these membranes exhibited both excellent and stable Rhodamine B (RhB) adsorption performance and removal capacity.

Rychtowski et al. [28] prepared TNR@Ni-foam structures via the alkaline hydrothermal method, which uses TiO_2_ with two different structures. In situ FTIR spectroscopy was used to assess the photodegradation properties of acetaldehyde in the TNR@Ni-foam structures. The results demonstrated that P25 facilitated the binding of Na species, thereby contributing to the formation of a layered Na_2_Ti_3_O_7_ structure and Ni(OH)_2_ species. This process resulted in a reduction in the electron transfer barrier and an increase in the separation of charge carriers, consequently achieving efficient photocatalytic acetaldehyde degradation.

Wang et al. [29] prepared n^+^-poly-Si layers with thicknesses ranging from 30 to 100 nm via low-pressure chemical vapor deposition (LPCVD). The thickness of the n^+^-poly-Si layer substantially suppressed metallization-induced recombination under metal interface contact, as well as on the contact resistivity of the cells. To circumvent the detrimental effects of Ag particle corrosion, low passivation metal values and resistivity under metal contact were ensured by optimizing the thickness of the n^+^-poly-Si layer to 70 nm with a surface atomic concentration of 5 × 10^20^/cm^3^. This optimization reduces the cost of n^+^-poly-Si layers for commercial applications in the photovoltaic industry.

Perovskite solar cell (PSC) stability can be improved through reducing the number of interfacial defects present at the interface between the perovskite and the electron transport layer. Du et al. [30] mixed two self-assembled molecules with different functional groups to form SnO_2_/perovskite interlayers, where -H_2_PO_3_ and -COOH improved the anchoring and carrier transport at the buried interface, respectively, as well as enhancing the photovoltaic performance. Furthermore, the amine group (-NH_2_) of the two small molecules has been shown to interact effectively with the uncoordinated Pb_2_^+^ in the perovskite layer, thereby enhancing the quality of the perovskite film and potentially reducing interfacial defects. This complementary synergistic passivation strategy has been demonstrated to enhance the stability of PSCs while concomitantly increasing their power conversion efficiency.

Selective emitter (SE) technology substantially influences the passivation and contact performance of n-TOPCon solar cells. To achieve the objective of clean production without the introduction of nitrogen, SiO_x_ is the optimal choice for large-scale production. n-TOPCon solar cells with different back-side phosphorus (P)-SE structures were prepared by Liu et al. [31] via three- and four-step methods, respectively. A comparison revealed that the four-step method exhibited higher performance and stability than that of the three-step method. However, with respect to full-scale electrical properties, both methods could yield results comparable results. This is highly important for improving the backside technology of n-TOPCon solar cells in mass production, improving the efficiency of solar cells and lowering energy usage.

Sn_3_O_4_ has emerged as a promising semiconductor material due to its excellent visible light absorption properties. Gribov et al. [32] prepared Sn_3_O_4_, SnO_2_ and Sn_3_O_4_/SnO_2_ using a hydrothermal method and investigated the photo-electrooxidation of a sequence of organic substrates with RHE under UV and visible light irradiation over a range of potentials from 0.6 to 1.4 V. The Sn_3_O_4_ exhibited a high level of activity in photo-electrooxidation reactions involving acetone and formic acid under visible-light conditions. The photo-electrooxidation of these substances exhibited high activity, whereas Sn_3_O_4_/SnO_2_ only demonstrated significant activity in formic acid oxidation. The occurrence of SnO_2_ particles within the Sn_3_O_4_/SnO_2_ composite enhances the photocurrent when subjected to UV irradiation. However, this phenomenon is accompanied by a substantial decline in oxidation efficiency under visible-light conditions.
